# Contribution of constitutively proliferating precursor cell subtypes to dentate neurogenesis after cortical infarcts

**DOI:** 10.1186/1471-2202-11-146

**Published:** 2010-11-17

**Authors:** Silke Keiner, Josephine Walter, Julia Oberland, Christoph Redecker

**Affiliations:** 1 Hans-Berger-Clinic for Neurology, University Hospital Jena, Erlanger Allee 101, D - 07747 Jena, Germany

## Abstract

**Background:**

It is well known that focal ischemia increases neurogenesis in the adult dentate gyrus of the hippocampal formation but the cellular mechanisms underlying this proliferative response are only poorly understood. We here investigated whether precursor cells which constitutively proliferate before the ischemic infarct contribute to post-ischemic neurogenesis. To this purpose, transgenic mice expressing green fluorescent protein (GFP) under the control of the nestin promoter received repetitive injections of the proliferation marker bromodeoxyuridine (BrdU) prior to induction of cortical infarcts. We then immunocytochemically analyzed the fate of these BrdU-positive precursor cell subtypes from day 4 to day 28 after the lesion.

**Results:**

Quantification of BrdU-expressing precursor cell populations revealed no alteration in number of radial glia-like type 1 cells but a sequential increase of later precursor cell subtypes in lesioned animals (type 2a cells at day 7, type 3 cells/immature neurons at day 14). These alterations result in an enhanced survival of mature neurons 4 weeks postinfarct.

**Conclusions:**

Focal cortical infarcts recruit dentate precursor cells generated already before the infarct and significantly contribute to an enhanced neurogenesis. Our findings thereby increase our understanding of the complex cellular mechanisms of postlesional neurogenesis.

## Background

The birth of new neurons in the adult brain takes place in discrete regions, especially in the dentate gyrus of the hippocampal formation. Following brain insults like stroke, this neurogenic region consistently boosts the generation of newborn neurons [1-4], but the mechanisms and functional role of this complex cellular response is only poorly understood. The subgranular zone (SGZ) of the dentate gyrus accommodates distinct precursor cell subtypes: radial glial-like type 1 cells (or B cells corresponding to the classification of Seri et al.) give rise to type 2 neuronal progenitors (D cells) which undergo selection and maturation into functional neurons (G cells) [5,6]. These cell types differ in their morphology, proliferative activity, migratory behaviour and expression of different key marker antigens [7]. Type 1 cells show a characteristic morphology with a triangle-shaped soma, long and strong apical processes reaching into the granular cell layer and astrocytic properties. Those radial glia-like cells express precursor cell markers like nestin and additional the astrocytic marker glial fibrillary acidic protein (GFAP). Transient amplifying precursor cells (type 2 cells) arising from type 1 cells still express nestin but lack GFAP. Type 2 cells have plump, short processes orientated parallel to the subgranular zone. Afterwards the precursor cells lose their nestin expression and are only positive for doublecortin (DCX) comprising a transition from a proliferative stage to postmitotic immature neurons. The proliferative activity is significantly increased with the expression of DCX [8]. After exit from the cell cycle the terminal postmitotic differentiation occurs and the cells express markers of mature neurons. The survival of new neurons is determined during the first 3 weeks after their birth [9,10] and is strongly regulated by neuronal network activity [11,12]. Neurons that have survived this period are incorporated into the hippocampal network [10]. In a previous study we demonstrated that the proliferation of distinct precursor cells in the dentate gyrus increases after focal cortical infarcts [2,13].

**Figure 1:**
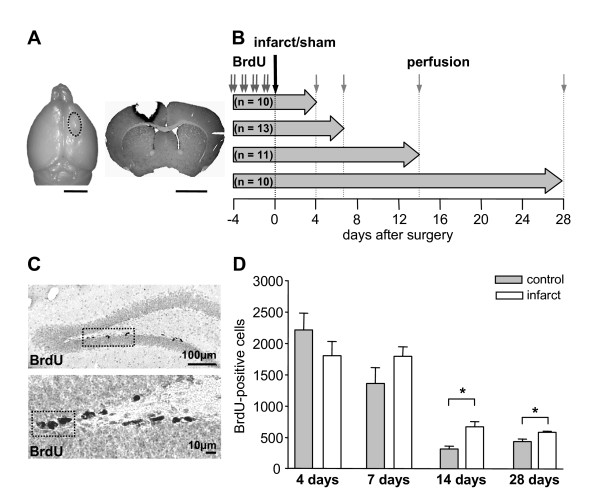
**Infarct model, experimental design and quantification of the number of bromodeoxyuridine (BrdU)-positive cells in the subgranular zone**. (A) Location and morphology of a photothrombotic infarct in the sensorimotor cortex on the brain surface (dotted line) and on a coronal BrdU-stained section at day 4 postsurgery. Scale bars represent 5 mm. (B) Schematic illustration of the experimental design. Prior to surgery all animals received intraperitoneal injections of the proliferation marker BrdU twice daily for 4 consecutive days and survived till day 4, 7, 14 or 28. (C) Immunohistochemically stained sections with antibodies against BrdU through the dentate gyrus of the hippocampus (two magnifications). (D) Diagram of the total number of BrdU-positive cells in the subgranular zone of the dentate gyrus at day 4, 7, 14 and 28. BrdU-positive cells significantly increase at day 14 and 28 postsurgery. Error bars represent S.E.M.. Significant differences (P < 0.05) are indicated by an asterisk.

In the present study we further analyzed whether precursor cells constitutively proliferating prior to infarct induction contribute to post-ischemic neurogenesis in the dentate gyrus. Therefore, the proliferation marker BrdU was injected before a focal ischemic infarct was photochemically induced in the forelimb sensorimotor cortex of adult transgenic nestin-GFP mice (Figure 1A, B). These transgenic mice, expressing GFP under the nestin promoter allow a clear identification of distinct nestin-positive precursor subpopulations [8,14-17]. Using this approach, we demonstrate that focal cortical infarcts sequentially increase newly generated precursor cell subtypes compared to sham-operated controls. We thereby provide evidence that hippocampal precursor cells already generated before the infarct contribute to post-ischemic neurogenesis.

## Results

All photothrombotically lesioned animals (n = 23) had typical cortical infarcts located in the sensorimotor cortex according to Paxinos [18] (Figure 1A). The infarcts impaired all cortical layers leaving the subcortical white matter intact. No structural lesions were observed in sham-operated controls (n = 21).

To study the recruitment of proliferative cells by cortical infarcts young adult animals received injections of the proliferation marker BrdU at 4 consecutive days prior to the induction of focal cortical infarcts or sham surgery (Figure 1B). The number of BrdU-labelled cells was quantified at day 4, 7, 14 and 28 post surgery in the subgranular zone (Figure 1B, C). In both experimental groups the number of BrdU-positive cells declined from day 4 (lesion: 1794 ± 225; control: 2201 ± 269) reaching 20 to 32% at day 28 after surgery (Figure 1D). The slight, but significant increase in number of BrdU-positive cells in control animals from day 14 to day 28 is probably caused by unspecific group differences due to a relatively small group size of 5 animals. Quantification of BrdU-positive cells revealed no significant differences between lesioned and sham-operated animals at day 4 and day 7. But we detected an increased survival of BrdU-positive cells at day 14 (lesion: 659 ± 86; control: 306 ± 47; p = 0.004) as well as at day 28 (lesion: 571 ± 20; control: 435 ± 27; p = 0.01) post infarct compared to controls (Figure 1D).

**Figure 2:**
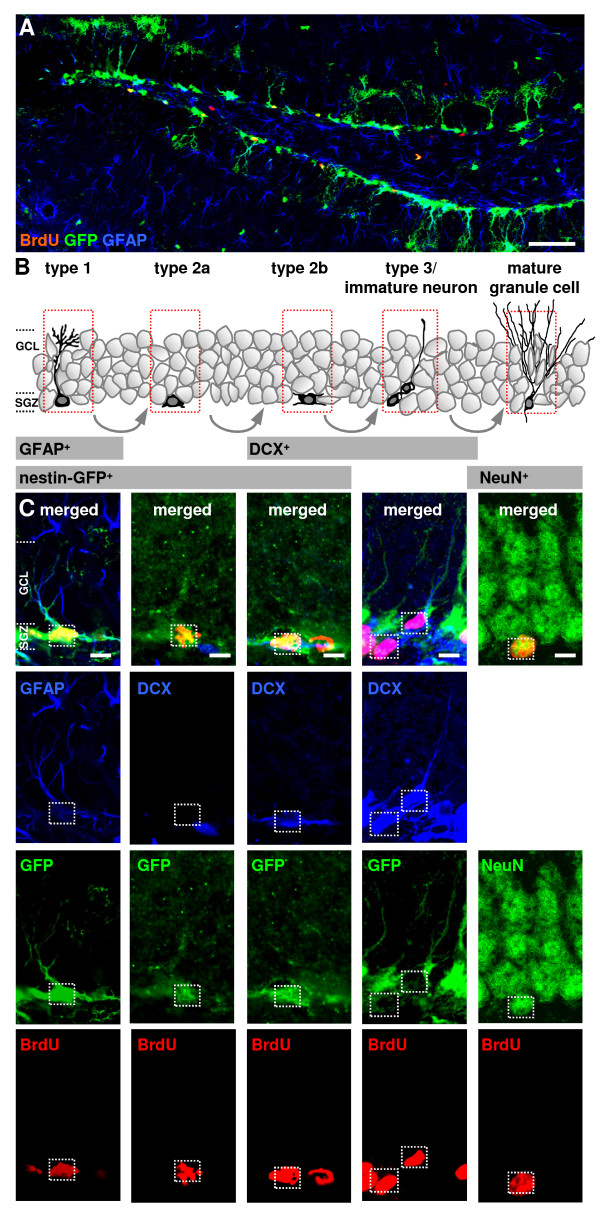
**Overview and distribution of distinct precursor cells in the dentate gyrus of the transgenic nestin-GFP mouse**. (A) labelled for bromodeoxyuridine (BrdU; red), green fluorescent protein (GFP; green) and glial fibrillary protein (GFAP; blue). Scale bar represent 100 μm. (B) Schematic illustration of distinct precursor cell subtypes leading to new neurons in the dentate gyrus. Type 1 cells exhibit characteristic morphology with a triangle-shaped soma, long and strong apical processes reaching into the granular cell layer (GCL). These radial glia-like cells express precursor cell markers like nestin and additionally the astrocytic marker glial fibrillary protein (GFAP). Arising from type 1 cells transient amplifying progenitor cells (type 2) express nestin, have plump, short processes orientated parallel to the subgranular zone (SGZ). They do not express GFAP. Type 2 cells exist in two subtypes, one negative (type 2a) and one positive for doublecortin (DCX) (type 2b). While type 2 cells express the marker nestin, type 3 cells are only positive for doublecortin and comprise a transition from a proliferative stage to postmitotic immature neurons. After exit from the cell cycle the terminal post mitotic differentiation of granular cells start the NeuN marker expression for mature neurons. (C) Confocal images of double- and triple-labelled immunofluorescent sections showing the distinct cell types (marked with dotted rectangles). Scale bars represent 10 μm. SGZ, subgranular zone; GCL, granular cell layer.

Furthermore we analyzed whether cortical infarcts affect distinct precursor cell populations which already took up BrdU prior to the infarct. Triple-immunocytochemistry with antibodies against BrdU, GFP and GFAP or DCX revealed four types of mitotically active precursor cell populations [2,8,19] corresponding to the classification described by Kempermann and colleagues [7] (Figure 2A, C). Type 1 cells express GFAP and nestin-GFP and generate nestin-GFP-positive type 2 cells lacking GFAP. Type 2 cells exist in two subtypes, one negative (type 2a) and one positive for DCX (type 2b). Type 2b cells divide into type 3 cells which are only positive for DCX. Newborn mature granule neurons were identified using antibodies against BrdU and NeuN.

**Figure 3:**
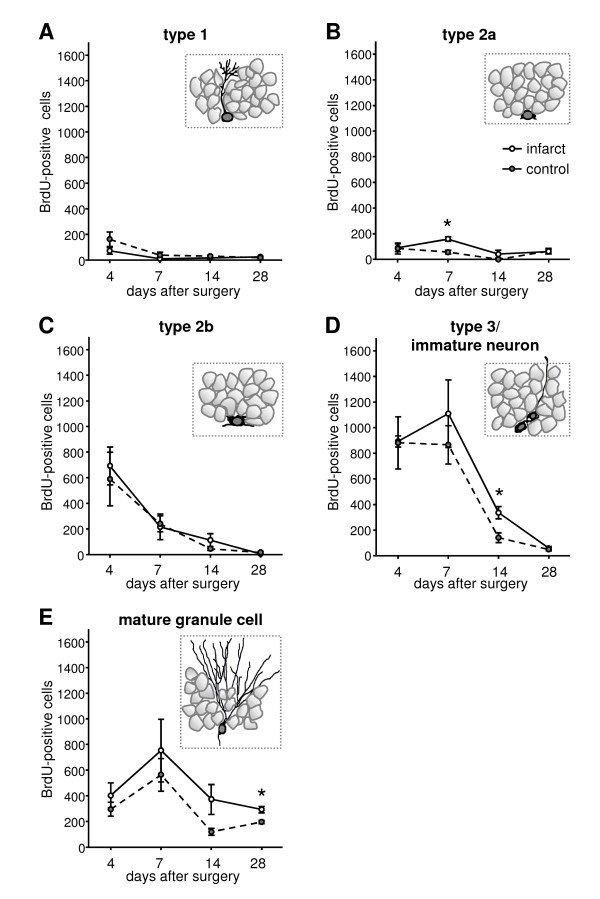
**Quantification of BrdU-positive precursor cell subtypes and newborn mature neurons in the dentate gyrus at day 4, 7, 14 and 28 after surgery**. Note the increase of BrdU-labelled type 2a cells at day 7, type 3/immature neurons at day 14 and mature neurons at day 28 after focal infarcts. Error bars represent S.E.M.. Significant differences (P < 0.05) between the experimental groups are indicated by an asterisk.

Quantification of these distinct cell types revealed a similar time-course for both experimental groups at day 4, 7, 14 and 28 post surgery (Figure 3A-E). Moreover, after focal cortical infarcts we detected a sequential increase of BrdU-positive type 2a cells at day 7, of type 3 cells/immature neurons at day 14 and of NeuN-positive cells at day 28 (Figure 3B, D, E). At day 4 post surgery the numbers of the precursor cell populations were similar in lesioned and sham-operated animals (Figure 3A-E). DCX-positive type 2b (lesion: 692 ± 148; control: 590 ± 209) and type 3 cells/immature neurons (lesion: 893 ± 43; control: 882 ± 204) exhibited the highest numbers of the BrdU-expressing cells (Figure 3C, D). The number of BrdU-positive type 2b cells decreased at day 7 (lesion day 4 to day 7: p = 0.03) whereas newborn type 3 cells/immature neurons remained on the same level and mature neurons increase (Figure 3C-E). At this time point the number of newborn type 2a cells showed a 3-fold enhancement in lesioned animals compared to controls (lesion: 159 ± 18; control: 56 ± 14; p = 0.01; Figure 3B). From day 7 the numbers of BrdU-positive type 2b, type 3/immature neurons (lesion day 7 to day 14: p = 0.03; day 14 to day 28: p = 0.004; control day 7 to day 14: p = 0.01) and mature neurons decrease in both groups (Figure 3C-E). But cortical infarcts increase the number of newborn type 3 cells/immature neurons at day 14 (lesion: 337 ± 48; control: 142 ± 38; p = 0.032) and mature neurons at day 28 (lesion: 293 ± 24; control: 196 ± 13; p = 0.038) compared to controls (Figure 3D, E). In contrast to the developmentally later precursor subtypes and mature neurons, the BrdU-expressing type 1 and type 2a cells revealed low numbers at all experimental time points in lesioned and control mice (Figure 3A, B).

**Figure 4:**
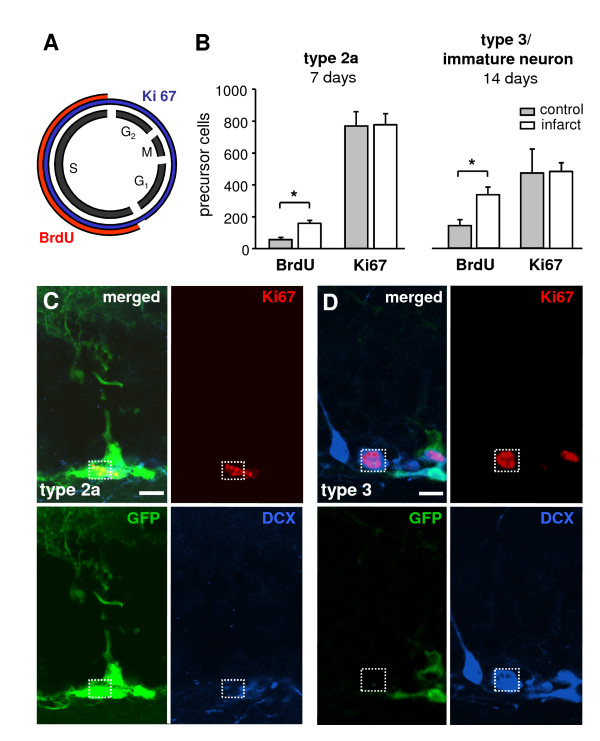
**Expression of different proliferation markers in type 2a cells at day 7 and type 3 cells/immature neurons at day 14 after surgery**. (A) Schematic illustration of the expression of the proliferation markers BrdU (red) and Ki67 (blue) during the cell cycle. (B) Quantification of BrdU- and Ki67-positive type 2a cells at day 7 and type 3 cells/immature neurons at day 14. Error bars represent S.E.M.. Significant differences (P < 0.05) are indicated by an asterisk. (C, D) Confocal images of Ki67-expressing type 2a (C) and type 3 cells (D). Scale bars represent 10 μm. GFP: green fluorescent protein, DCX: doublecortin

In order to analyze whether changes in proliferative activity of type 2a cells and type 3 cells/immature neurons contribute to the increase of newborn neurons, we additionally stained for Ki67 as an endogenous proliferation marker (Figure 4A, C). Using this approach we detected no differences in number of Ki67-expressing type 2a cells at day 7 and type 3 cells/immature neurons at day 14 in animals with cortical infarcts and sham-operated controls (Figure 4B). These data support our observation that the infarct favours the differentiation and survival of precursor cells constitutively proliferating prior to the insult.

Taken together, our findings demonstrate that focal cortical infarcts affect precursors generated already before the infarct and significantly enhance their survival.

## Discussion

The present findings support our previous reports that small cortical infarcts increase neurogenesis in the young dentate gyrus mainly by promoting the survival of newborn cells [20,21]. Several studies already demonstrated that focal ischemia stimulates the proliferation of dentate precursor cells after infarct induction [2,19,22]. We here provide novel data that precursor cells which constitutively divide prior to the infarct significantly contribute to post-lesional neurogenesis. This finding is in agreement with a previous study showing that epileptic activity stimulates dentate precursor cells actively proliferating before the insult [23]. In this study, BrdU-labelling one day prior to seizure induction resulted in increased numbers of BrdU-positive cells at days 4 to 10 and led to more immature neurons at days 7 to 14 after injury. The time-course of this proliferative response was only slightly delayed after focal cortical infarcts, probably due to different lesion mechanisms. However, large infarcts induced by occlusion of the middle cerebral artery enhance precursor cell proliferation 4 to 7 days after the lesion [24-26].

In agreement with Kempermann and colleagues [9] we found a non linear decline of BrdU-positive cells in our control animals. This group described a survival of approximately 20% of BrdU-expressing cells 4 weeks after a period of BrdU-injections compared to day 1 which is in accordance with our study. The decrease of BrdU-DCX-expressing cells in controls from day 4 until day 28 post surgery (5 to 29 days after the last BrdU-injection) in our study is also consistent with a report from Brown and colleagues [27] indicating a similar decline mainly between 7 and 30 days after BrdU-injections. Additionally the proportion of mature neurons among BrdU-positive cells 4 weeks after BrdU-labelling in a previous report [28] (54%) is similar to our findings (45 ± 5%).

The numbers of newborn precursor cell subtypes increase sequentially after the infarct with exception of type 2b cells which were not enhanced at any time point. Type 2a cells give rise to type 2b cells which than generate type 3 cells. Cortical infarcts increased the number of newborn type 2a cells at day 7, enhanced type 3 cells/immature neurons at day 14 and mature neurons at day 28 after infarcts. We further demonstrate that the enhancement of the newborn mature neurons is mainly caused by an enhanced differentiation and survival of precursor cells born already before the infarct since we could not detect any changes in proliferative activity of these distinct cell types. It is possible that we missed a change in the number of type 2b cells. Additionally the absolute number and proliferation rate of the precursor cells might change after infarcts leading to an alteration of type 2b cells which was not detected in our study. We previously demonstrated that proliferation rates and absolute numbers of distinct precursor cell populations differ under physiological conditions [8]. Type 1 and type 2a cells exhibit higher absolute cell numbers but fewer dividing cells compared to later precursor cell populations.

The mechanisms of post-ischemic neurogenesis are still only poorly understood. Glutamatergic mechanisms have been shown to be involved in ischemia-mediated neurogenesis [1,20]. Also interruption of neuronal pathways by stroke may provide a trigger for neurogenesis because induction of hippocampal seizure activity by unilateral perforant path stimulation bilaterally increased dentate neurogenesis [29]. Recent studies provide evidence that unilateral lesions of the entorhinal cortex, the main excitatory input of granule cells, do not alter the proliferation in the dentate gyrus but bilaterally enhance the survival of newborn neurons [30,31]. This bilateral increase was also detected in several previous studies using the same model for ischemia as in the present study [2,21]. Furthermore, diffusible and humoral factors might contribute to the neurogenic response in the dentate gyrus [32-34]. The activation of the transcription factor cAMP response element-binding protein (CREB) seems to play an important role for the survival of newborn neurons in the dentate gyrus under physiological conditions [35] as well as after ischemia [36,37]. Moreover, N-methyl-D-aspartate-type glutamate receptor-mediated network activity contributes to the selective survival of newborn neurons [11].

To elucidate the functional role of newborn neurons in the dentate gyrus a number of hypotheses have been developed [38-42]. It remains still unexplained why the adult brain responds to insults like stroke with an increased neurogenesis in the dentate gyrus. Moreover, increased levels of newborn granule cells generated in the dentate gyrus after stroke correlate with better functional outcomes in several models of focal ischemia when the animals received different types of rehabilitative training [21,43-45].

## Conclusions

Taken together, the present study provides evidence that focal cortical infarcts affect hippocampal precursor cells, constitutively proliferating prior to infarct induction and enhance their survival. These alterations significantly contribute to increased neurogenesis and elucidate a new aspect of the complex neurogenic response of the dentate gyrus after brain ischemia.

## Methods

### Induction of photothrombotic infarcts

The present study was performed in accordance with the guidelines of the National Institutes of Health (revised 1987) and all experimental procedures were approved by the German Animal Care and Use Committee. Photothrombotic infarcts initially described by Watson et al. [46] were induced in the sensorimotor cortex (Figure 1A) [18] on a total number of 23 male transgenic mice (age: 12 - 14 weeks) expressing GFP under the control of nestin promoter [17]. Briefly, animals were anesthetized with 2.5 - 2.0% isoflurane and 30% oxygen. A fiberoptic bundle (1.3 mm diameter) connected to the cold light source (KL 1500, Schott, Jena, Germany) was positioned on the skull 0.4 mm anterior relative to bregma and 2.0 mm lateral to the midline [18]. Rose Bengal (1.3 mg/100 mg body weight) dissolved in saline (1 ml/100 μl) was injected intraperitoneal 5 min before illumination (duration 15 min). The beam had a light intensity of 15 W/cm^2 ^and the color temperature was 3200 K. 21 animals served as control receiving the same treatment without illumination. All animals were housed in standard cages under 12 h light/12 h dark conditions with free access to food and water.

### BrdU injections and tissue processing

Prior to photothrombotic infarct or sham surgery all animals received intraperitoneal injections of the proliferation marker BrdU 50 mg/kg (Sigma-Aldrich, Taufkirchen, Germany) dissolved in 0.9% saline for 4 consecutive days twice per day 8 h apart. Animals were allowed to recover for 4, 7, 14 or 28 days after infarct induction. The animals were deeply anesthetized with diethylether and perfused transcardially with 4% phosphate buffered paraformaldehyde. Brains were removed immediately after perfusion and postfixed in 4% paraformaldehyde overnight. For cryoprotection the tissue was transferred into 10% sucrose for 24 h and 30% sucrose for 48 h and stored at -75 °C for further processing. The experimental design and BrdU injection protocol are illustrated in Figure 1B.

### Immunocytochemistry

Sequential sections were cut into 40 μm with a freezing microtome and collected in 0.1 mol/l phosphate-buffered saline. For immunocytochemistry staining, free-floating sections were treated for 30 min with 0.6% H_2_O_2 _in Tris-buffered saline (TBS; pH 7.5) to block endogenous peroxidase. After washing sections were denaturised for 30 min in 2 N HCl at 37 °C followed by rinsing in boric acid (10 min, pH 8.5). After several rinses in TBS (containing 0.25% Triton X-100) the sections were incubated overnight at 4 °C in primary rat monoclonal anti-BrdU (1 : 500) containing 3% normal donkey serum and 0.1% Triton X-100 in TBS. Next day the sections were rinsed in TBS, blocked for 30 min (containing 0.25% Triton X-100 and 3% normal donkey serum) and incubated in the secondary antibody (biotinylated donkey anti-rat antisera, 1 : 500, Jackson Immunoresearch, West Grove, PA) for 2 h at room temperature. The slices were then treated in avidin-biotin-peroxidase complex for 60 min (Vector Laboratories, Burlingame, CA, USA). Furthermore the sections were incubated into a solution of 3.3-diaminobenzidine (0.25 mg/ml; Sigma-Aldrich, Munich, Germany) containing 0.01% H_2_O_2_. After peroxidase staining the sections were washed, mounted and coverslipped (Entellan; Merck, Darmstadt, Germany).

BrdU-positive precursor cell subtypes were immunocytochemically identified by triple-labelling of BrdU, GFP and GFAP; BrdU, GFP and DCX and newborn neurons by double-labelling of BrdU and NeuN. Ki67-expressing precursor cell subtypes (type 2a at day 7, type 3 at day 14) were immunocytochemically identified by triple-labelling of Ki67, GFP and DCX. For immunofluorescence staining the slices were treated in the same way described previously for the immunoperoxidase staining. Sections were treated for 24 h with the primary antibodies rat anti-BrdU (1 : 500; Immunologicals Direct-Oxford Biotechnology, Oxfordshire, UK), rabbit anti-Ki67 (1 : 250; Novocastra; Newcastle upon Tyne, U.K.), mouse anti-GFP (1 : 500; Santa Cruz, CA, USA), guinea pig anti-GFAP (1 : 500; Advanced Immunochemistry, USA), rabbit anti-DCX (1 : 500 Cell Signalling Technology, Danvers, USA), goat anti-DCX (1 : 200; Santa Cruz, CA, USA) and mouse anti-NeuN (1 : 500, Chemicon, Temecula, CA). After rinsing with TBS, 0.1% Triton X-100 and 3% donkey serum for 30 min, sections were incubated for 2 h in a mixture of TBS, 0.1% Triton X-100 and 3% donkey serum and the following secondary antibodies: Rhodamine anti-rat (1 : 500; Dianova, Hamburg, Germany), Rhodamin anti-rabbit (1 : 500, Dianova, Hamburg, Germany), CY5 anti-guinea pig (1 : 500; Dianova, Hamburg, Germany), CY5 anti-rabbit (1 : 500; Dianova, Hamburg, Germany), CY5 anti-goat (1 : 500; Dianova, Hamburg, Germany), Alexa Fluor 488 anti-mouse (1 : 500; Molecular Probes, Leiden, Netherlands) at room temperature. Subsequent the sections were rinsed in TBS and coverslipped with Mowiol 40-88-DABCO medium (Sigma-Aldrich, Taufkirchen, Germany).

### Quantification and confocal microscopy

The quantification of the peroxidase-stained BrdU-positive cells and immunofluorescent labelled Ki67-expressing cells were performed with an Axioplan two imaging microscope (Carl Zeiss AG, Jena, Germany) using a 40× magnification. One in six series of sections throughout the rostrocaudal extent of the ipsi- and contralateral subgranular zone were counted. To obtain the estimated total number of the BrdU-positive cells the resulting numbers were multiplied by the factor 6. The phenotype analysis of the BrdU-and Ki67-labelled cells was performed with a confocal laser scanning microscope (LSM 710, Carl Zeiss AG, Jena; Germany). For characterisation 20 to 100 BrdU-labelled cells were determined per staining in 4 - 6 animals by co-localisation of BrdU, GFP and GFAP (type 1 cells) or BrdU, GFP and DCX (type 2a, type 2b, type 3 cells/immature neurons) as well as BrdU and NeuN (mature neurons). This co-localisation was carried out for both dentate gyri and was confirmed by z-series through the cell soma allowing the definite assessment of overlap between the antigens. To assess the proliferative response of type 2a cells at day 7 (control: n = 3; infarcted: n = 3) and type 3 cells at day 14 (control: n = 4; infarcted: n = 4) the phenotype of 35 to 70 Ki67-positive cells per animal were determined in the SGZ of both hemispheres of every 12^th ^section. The absolute number of newly generated distinct subpopulations in the subgranular zone was calculated per animals by multiplying the absolute number of BrdU-and Ki67-positive cells with the percentage of the corresponding phenotype.

### Statistical analysis

All statistical analyses were performed with SPSS for Window 11.5 (Standard Version). We first tested for normal distribution using the Shapiro-Wilk test. Statistical significance differences for the cell counts and phenotype analysis were assessed using the Mann Whitney-U-test. All analysis results are expressed as mean ± SEM. A level of P < 0.05 was considered statistically significant.

## Authors' contributions

SK conceived the study and designed the experiments, analyzed the findings and wrote the manuscript; JO carried out the experiments; JW acquired the data and analyzed the findings and wrote the manuscript; CR designed the study and helped to draft the manuscript. All authors read and approved the final manuscript.
